# BK channels: multiple sensors, one activation gate

**DOI:** 10.3389/fphys.2015.00029

**Published:** 2015-02-06

**Authors:** Huanghe Yang, Guohui Zhang, Jianmin Cui

**Affiliations:** ^1^Ion Channel Research Unit, Duke University Medical CenterDurham, NC, USA; ^2^Department of Biochemistry, Duke University Medical CenterDurham, NC, USA; ^3^Department of Biomedical Engineering, Washington University in Saint LouisSt. Louis, MO, USA; ^4^Cardiac Bioelectricity and Arrhythmia Center, Washington University in Saint LouisSt. Louis, MO, USA; ^5^Center for The Investigation of Membrane Excitability Disorders, Washington University in Saint LouisSt. Louis, MO, USA

**Keywords:** BK channels, allosteric gating, calcium binding proteins, modular organization, ion permeation, voltage sensor domain, magnesium binding, ion channel gating

## Abstract

Ion transport across cell membranes is essential to cell communication and signaling. Passive ion transport is mediated by ion channels, membrane proteins that create ion conducting pores across cell membrane to allow ion flux down electrochemical gradient. Under physiological conditions, majority of ion channel pores are not constitutively open. Instead, structural region(s) within these pores breaks the continuity of the aqueous ion pathway, thereby serves as activation gate(s) to control ions flow in and out. To achieve spatially and temporally regulated ion flux in cells, many ion channels have evolved sensors to detect various environmental stimuli or the metabolic states of the cell and trigger global conformational changes, thereby dynamically operate the opening and closing of their activation gate. The sensors of ion channels can be broadly categorized as chemical sensors and physical sensors to respond to chemical (such as neural transmitters, nucleotides and ions) and physical (such as voltage, mechanical force and temperature) signals, respectively. With the rapidly growing structural and functional information of different types of ion channels, it is now critical to understand how ion channel sensors dynamically control their gates at molecular and atomic level. The voltage and Ca^2+^ activated BK channels, a K^+^ channel with an electrical sensor and multiple chemical sensors, provide a unique model system for us to understand how physical and chemical energy synergistically operate its activation gate.

## Introduction

**Figure 1:**
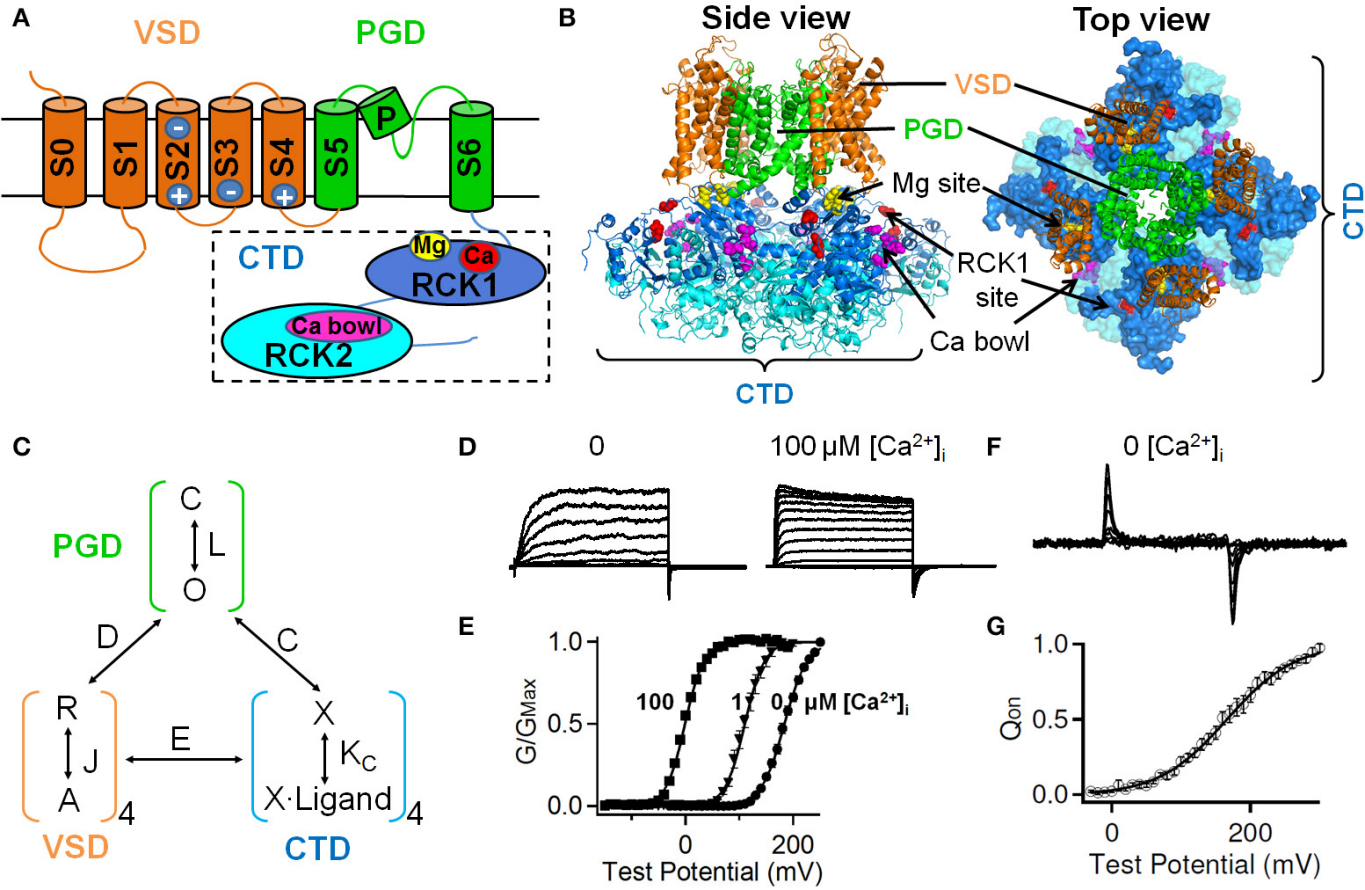
**Structural domains of the large-conductance, Ca^2+^- and voltage-activated BK channels and their allosteric interactions during channel gating**. **(A)** A BK channel can be divided into three structure domains: the pore-gate domain (PGD), the voltage sensor domain (VSD) and the cytosolic tail domain (CTD). Major elements for voltage and ion sensing (see text) are illustrated. **(B)** A homology model of BK channels based on the CTD structure of the zebra fish BK channel (PDB ID: 3U6N) and the membrane spanning domain of the Kv1.2–Kv2.1 chimera channel structure (PDB ID: 2R9R) by superimposing to the corresponding conserved regions of the MthK channel structure (PDB ID: 1LNQ) using UCSF Chimera software. Different structural domains are shown in colors as in A. **(C)** A general allosteric gating mechanism including allosteric interactions among three structure domains. *C* and *O*: closed and open conformations of PGD, respectively; *L*: the equilibrium constant for the *C*–*O* transition in the absence of voltage sensor activation and Ca^2+^ binding; R and A: resting and activated states of VSD; *J*: the equilibrium constant for VSD activation; *K*_*C*_: equilibrium constant for ligand binding to closed channels; *D, C, and E*: allosteric factors describing the interaction between PGD-VSD, PGD-CTD, and VSD-CTD, respectively. **(D)** Macroscopic ionic current of BK channels in the absence and presence of 100 μM [Ca^2+^]_i_. **(E)** Increasing [Ca^2+^]_i_ shifts the conductance-voltage (*G–V*) relation to more negative voltages. **(F,G)** In the absence of Ca^2+^, VSD can move in response to membrane voltage changes. **(F)** Gating current traces. Gating currents are generated due to the movement of the voltage sensor in the electric field across the membrane. **(G)** The voltage dependence of gating charge movement, the *Q–V* relation.

BK channels, also known as MaxiK, Slo1 or K_Ca_1.1 channels, are one type of calcium-activated potassium channels that have large single channel conductance of 100–300 pS (Marty, 1981; Pallotta et al., 1981; Latorre et al., 1982). As a member of the six transmembrane (TM) voltage-gated potassium (K_V_) channel superfamily, the basic functional unit of BK channels is a tetramer of the pore-forming *α*-subunits encoded by *Slo1* or *KCNMA1* gene in human (Figure 1A). The *slo1* gene was first identified by studying a mutation of the *Drosophila Slowpoke* locus that specifically abolished a Ca^2+^-activated K^+^ current in fly muscles and neurons (Atkinson et al., 1991; Adelman et al., 1992). BK channel activation can be regulated by membrane voltage and various intracellular chemical ligands such as Ca^2+^ (Marty, 1981; Pallotta et al., 1981; Adams et al., 1982; Barrett et al., 1982; Latorre et al., 1982; Methfessel and Boheim, 1982; Moczydlowski and Latorre, 1983), Mg^2+^ (Squire and Petersen, 1987; Zamoyski et al., 1989; Ferguson, 1991; McLarnon and Sawyer, 1993; Zhang et al., 1995, 2001; Morales et al., 1996; Wachter and Turnheim, 1996; Bringmann et al., 1997; Shi and Cui, 2001; Shi et al., 2002; Xia et al., 2002), protons (Schubert et al., 2001; Avdonin et al., 2003; Brelidze and Magleby, 2004; Hou et al., 2009), heme (Tang et al., 2003; Horrigan et al., 2005), carbon monoxide (Williams et al., 2004, 2008; Hou et al., 2008a), ethanol (Jakab et al., 1997; Dopico et al., 1998; Davies et al., 2003; Liu et al., 2008c; Bukiya et al., 2014; Davis et al., 2014), and lipid molecules (Braun, 2008; Vaithianathan et al., 2008; Yuan et al., 2011; Bukiya et al., 2011b; Dopico et al., 2012; Latorre and Contreras, 2013; Hoshi et al., 2013b,c,d; Tang et al., 2014) (Figures 1A,B, **3** and Table 1). The properties of BK channels can be further diversified through various splicing variants (Tseng-Crank et al., 1994; Navaratnam et al., 1997; Rosenblatt et al., 1997; Fury et al., 2002), post-translational modifications (Schubert and Nelson, 2001; Li et al., 2010), and association with the tissue-specific auxiliary β (Tseng-Crank et al., 1996; Wallner et al., 1996; Behrens et al., 2000; Orio et al., 2002) and γ subunits (Yan and Aldrich, 2010, 2012). Owing to their big conductance, the opening of BK channels allows rapid efflux of potassium ions, which effectively hyperpolarizes membrane potential, regulates membrane excitability, intracellular ion homeostasis, calcium signaling and cell volume. Therefore, BK channels are important in controlling various physiological processes, including smooth muscle contraction (Brayden and Nelson, 1992; Nelson et al., 1995; Tanaka et al., 1998; Perez et al., 1999; Pluger et al., 2000; Wellman and Nelson, 2003), hormone secretion (Petersen and Maruyama, 1984; Wang et al., 1994; Ghatta et al., 2006; Braun et al., 2008), neural excitation (Adams et al., 1982; Lancaster and Nicoll, 1987; Storm, 1987; Roberts et al., 1990; Robitaille and Charlton, 1992; Robitaille et al., 1993), hearing (Hudspeth and Lewis, 1988b,a; Wu et al., 1995; Rosenblatt et al., 1997; Fettiplace and Fuchs, 1999), circadian rhythms (Meredith et al., 2006), and gene expression (Marty, 1981; Li et al., 2014a). Consistent with their important physiological roles, BK channels have been discovered involving in pathogenesis of various diseases such as epilepsy (Du et al., 2005; N'Gouemo, 2011), cerebellar ataxia (Sausbier et al., 2004), autism and mental retardation (Laumonnier et al., 2006; Deng et al., 2013), stroke (Gribkoff et al., 2001), hypertension (Brenner et al., 2000), asthma (Seibold et al., 2008), tumor progression (Weaver et al., 2004; Sontheimer, 2008), obesity (Jiao et al., 2011), hypoxia and ischemia (Kumar, 2007; Tano and Gollasch, 2014). With the collective efforts of the BK channel field, the understanding of molecular mechanisms of BK channel function has been greatly advanced over the past three decades. This review summarizes the recent structure-function understanding of the sensors and the activation gate of BK channels, their allosteric coupling, and implications of their assembly in 3-dimension. The readers may refer to other excellent reviews with regard to BK channel structure-function, physiology and regulations (Toro et al., 1998; Magleby, 2003; Cox, 2006; Latorre and Brauchi, 2006; Salkoff et al., 2006; Cui et al., 2009; Latorre et al., 2010; Lee and Cui, 2010; Horrigan, 2012; Rothberg, 2012; Singh et al., 2012b; Hoshi et al., 2013a; Yang and Cui, 2015) and reviews in this special topics series.

**Table 1:** Molecular nature of chemical sensors in BK channels^*^.

**Ligands**	**Effect**	**Binding site(s)**	**References**
Ca^2+^	Activation	D367, R514, and E535 (in RCK1) and Ca-bowl: D889, D892, D895, and D897 (in RCK2)	Schreiber and Salkoff, 1997; Shi et al., 2002; Xia et al., 2002; Zhang et al., 2010a
Mg^2+^	Activation	D99 and N172 (in VSD) + E374 and E399 (in RCK1)	Shi et al., 2002; Xia et al., 2002; Yang et al., 2008
H^+^	Activation	H365 and H394 (in RCK1)	Hou et al., 2008b
Carbon monoxide	Activation	Potentially through H365, D367, and H394 (in RCK1)	Hou et al., 2008a
Ethanol	Activation	K361 and R514 (in RCK1)	Bukiya et al., 2014
Heme	Inhibition	C_612_KAC_615_H_616_ (between RCK1 and RCK2)	Tang et al., 2003
PIP_2_	Stabilization?	K392, R393 (in RCK1)	Tang et al., 2014
Omega-3	Activation	Y318 (in S6)	Hoshi et al., 2013d

* *The residue numbers are according to the sequence of the mbr5 clone*.

## BK channel structure follows a modular design

A functional BK channel is comprised of four Slo1 subunits. Each Slo1 subunit has three main structural domains with distinct functions (Figure 1A). The pore-gate domain (PGD) opens and closes to control ion selectivity and K^+^ permeation; the voltage sensor domain (VSD) senses membrane potential changes; and the large cytosolic tail domain (CTD) that occupies two third of Slo1 sequence forms a gating-ring and serves as the chemical sensor to detect intracellular Ca^2+^ ions and various other ligands (Figures 1B, **3** and Table 1). Two sensory domains, the VSD and the CTD, covalently attach to the N- and C-terminus of the PGD, respectively. The basic function of these sensory domains is to transduce electric or chemical energy to mechanical forces on the PGD to toggle its conformation between closed and open states to control K^+^ flux (Figure 1C).

The three distinct structural domains in BK channels can work as functionally independent modules and their homologs are widely expressed in various organisms. The PGD of BK channel is homologous to the PGDs of numerous prokaryotic and eukaryotic 2-TM and 6-TM K^+^ channels, while the BK channel VSD follows similar design as the VSDs of voltage-gated cation channels (Long et al., 2005b,a, 2007; Payandeh et al., 2011), proton channels (Ramsey et al., 2006; Sasaki et al., 2006; Takeshita et al., 2014) and voltage sensitive phosphatase (VSPs) (Murata et al., 2005; Li et al., 2014b). Homologs of BK CTD have been found in the cytosolic domains of the bacterial K^+^ channel complex (Cao et al., 2013), prokaryotic ligand-gated K^+^ channels (Jiang et al., 2002a,b), as well as of the CTDs of Na^+^- and Cl^−^- activated Slo2 and pH-regulated Slo3 K^+^ channels (Schreiber et al., 1998; Yuan et al., 2003, 2010; Salkoff et al., 2006). Based on the sequence homology, it seems plausible that multiple lateral gene transfer and gene fusion events might have occurred during the evolution of BK channels to link all three individual modules to form a multi-functional ion channel. Consistent with this possibility, a recent study elegantly demonstrated that a truncated BK channel without the entire CTD specifically eliminates its capability to sense intracellular ligands; but the voltage sensing and K^+^ permeation are largely intact (Budelli et al., 2013). On the other hand, a prokaryotic MthK channel that is comprised of a PGD and a similar cytosolic gating-ring structure but lacks VSD is activated by intracellular Ca^2+^ (Jiang et al., 2002a,b).

The three distinct structural domains interact with one other and dynamically regulate channel gating, making BK channels an exemplar model system to study principles of sensor-gate coupling in ion channel function. Under physiological conditions, Ca^2+^ and depolarization work on the CTD and the VSD, respectively. The free energy derived from these two separate sensory modules activates the PGD of BK channels. The structure-function relationships of each individual module and the current understanding of their couplings are described below.

## The voltage sensor domain serves as the electric sensor of BK channels

Membrane depolarization alone is sufficient to activate BK channels as evidenced by the voltage-dependent macroscopic ionic current and the fast gating current that proceeds the ionic current in the absence of Ca^2+^ (Figures 1D–G). The voltage-dependence is mainly derived from voltage sensing residues in their intrinsic voltage sensor domain (VSD), which transverse membrane electrical field resulting in the measurable gating current (Figures 1F,G). The VSD of BK channels resembles a similar design to the VSDs of other voltage sensitive transmembrane proteins that include four transmembrane helices S1–S4. Unique to BK channel VSD, an additional transmembrane helix S0 (Meera et al., 1997) had been evolutionarily fused to its N-terminus through a long (~70 amino acids) intracellular loop (the S0–S1 linker), rendering the N-terminus of Slo1 peptide to the extracellular side (Figure 1A). Biochemical and electrophysiological evidence suggests that the extracellular end of S0 is located in close proximity of S3 and S4 (Liu et al., 2008a,b; Wang and Sigworth, 2009) and contributes to the folding and function of BK channel VSD (Meera et al., 1997; Koval et al., 2007; Pantazis et al., 2010b).

The VSD of BK channels exhibits three major functional differences from the VSDs of other Kv channels. First, BK channel VSD carries much less voltage-sensing charges (gating charge) than Kv channel VSDs (Stefani et al., 1997; Horrigan and Aldrich, 1999; Ma et al., 2006). The canonical Shaker K^+^ channel has ~12–13*e* effective gating charges (Zagotta et al., 1994; Aggarwal and MacKinnon, 1996; Seoh et al., 1996), whereas each VSD of a BK channel only carries 0.6*e* gating charge or 2.4*e* charges per channel. The smaller number of gating charges indicates that more membrane depolarization is needed to move the VSD of BK channels into the fully activated state, as evidenced by the shallower slope of the gating charge-voltage (*Q–V*) relationship in gating current measurement (Figure 1G) and the conductance-voltage (*G–V*) relationship in ionic current measurement (Figure 1E). This weaker voltage sensitivity is critical to the physiological role of BK channels because it enables BK channels to operate in a wide range of membrane potentials to fine-tune channel activation, and in turn the membrane voltage. Second, only one out of three Arginine residues in BK channel S4 contributes to gating charge. Mutations of the other two Arginine residues, R207 or R210, do not affect the total gating charge, while voltage-sensing R213 merely contributes 0.3*e* to each VSD (Ma et al., 2006). This is drastically different from the S4 of Kv channels, in which each of the first four Arginine (R1-R4) residues accounts for about 1*e* gating charge (Aggarwal and MacKinnon, 1996; Gandhi and Isacoff, 2002; Bezanilla, 2008). Third, the voltage sensing residues in BK channels are not restricted to S4. It has been well established that the R1-R4 residues in S4 serve as the primary voltage sensor of Kv channels and account for nearly all their gating charge (Aggarwal and MacKinnon, 1996). Nevertheless, BK channel S4 only contributes about half of total gating charge (Ma et al., 2006). In addition, E219, an acidic residue at the C-terminus of S4, was suggested to sense voltage (Zhang et al., 2014), bringing the contribution of S4 to total gating charge even lower. The other voltage sensing residues, D153 and R167 in S2 and D186 in S3 (Figure 1A), collectively contribute at least 50% of gating charge of BK channels. Interestingly, the corresponding residues in the Shaker K^+^ channel have minimal contribution to its gating charge (Seoh et al., 1996). Instead, the acidic residues corresponding to D153 and D186 in Kv channels have been shown to form a network of electrostatic interaction with arginine residues in S4 at either the resting or active state, thereby controlling the conformational stability of the VSD (Seoh et al., 1996; Tiwari-Woodruff et al., 1997; Silverman et al., 2003; Long et al., 2005a). On the other hand, E293, the only major voltage sensing residue in Shaker S2, corresponds to an uncharged residue in the BK channel (Y163) (Seoh et al., 1996). Even replacing Y163 with a glutamate residue did not enhance the voltage sensing of BK channels (Ma et al., 2006). The decentralized distribution of gating charges and small contribution of each voltage sensing residue to BK channel activation thus suggest that the VSD movement in BK channels during channel gating may differ from that in Kv channels (Ma et al., 2006). Consistent with this scenario, recent voltage clamp fluorometry studies demonstrated that the transmembrane helices in BK channel VSD undergo complex relative motions during voltage-dependent activation. Upon depolarization, S2 approaches S1, while S4 diverges from S0, S1, and S2 (Pantazis et al., 2010b; Pantazis and Olcese, 2012). The relative movements of the voltage sensing S2 and S4 segments in the membrane electrical field result in reciprocal and cooperative interactions between these two transmembrane segments as evidenced by the fact that the neutralization of voltage-sensing residues in one segment impairs the voltage-dependent motions of the other (Pantazis et al., 2010a). This cooperativity between S2 and S4 may derive from mechanical coupling between the two segments. Alternatively or additionally, this cooperativity may be mediated by the rearrangements of the aqueous crevices within the VSD, which can change the dynamic focusing of the membrane electric field.

## The cytosolic tail domain serves as the chemical sensor of BK channels

### Structures of the cytosolic tail domain (CTD)

The CTD of BK channel contains multiple ligand binding sites (**Figure 3**), serving as the primary chemical sensor to respond to changes of Ca^2+^ and other intracellular ligands. The main structural components of a CTD are two regulators of K^+^ conductance (RCK) domains (RCK1 and RCK2) that are connected by a ~100-amino acid linker (Figure 1A). Evolutionarily conserved in the CTDs of some eukaryotic and many prokaryotic ligand-gated K^+^ channels, as well as many prokaryotic K^+^ transport systems, RCK domains regulate K^+^ transport in response to intracellular ligand binding (Jiang et al., 2001, 2002a; Kuo et al., 2005; Loukin et al., 2005; Albright et al., 2006; Fodor and Aldrich, 2006). Recently, three crystal structures of eukaryotic BK CTD (PDB ID: 3MT5, 3U6N and 3NAF) were solved (Wu et al., 2010; Yuan et al., 2010). These structures provide the molecular basis for further understanding of how BK channel CTD regulate channel gating upon ligand binding.

The overall architecture and some key structural features are conserved between BK channel CTD and its prokaryotic counterparts. Four CTDs or eight RCK domains from a tetrameric BK channel stack together and form a large gating ring structure that covalently connects to the C-terminus of the PGD through four ~20-amino acid C-linker (Figure 1A). Nevertheless, BK channel gating ring structures exhibit unique features. Different from the prokaryotic MthK channel whose CTD contains two separate identical RCK domains (Jiang et al., 2002a), the BK CTD contains the tandem non-identical RCK1-RCK2 domains and assembles into a more expanded gating ring structure. Each RCK domain can be further divided into three subdomains: the Rossmann-fold subdomain (β A–β F) forms central core of the gating ring; the intermediate helix-crossover (αF-turn-αG) interlocks RCK1 and RCK2 domains within the same subunit; and the C-terminal subdomain (αH–C-terminus) stays in the periphery and helps to hold the integrity of the gating ring structure. Extensive inter-RCK interactions at the helix crossover and C-terminal subdomain result in a more extensive “flexible interface” within the same subunit; while the “assembly interface” is mainly restricted to the Rossmann-fold subdomains between neighboring subunits. Under physiological conditions, Ca^2+^ is the major BK channel regulator that binds to the CTD. CTD also interacts with VSD via intracellular Mg^2+^ to activate the channel. Other ligands such as protons, heme, phosphatidylinositol 4,5-bisphosphate (PIP_2_) and ethanol also bind to the CTD and regulate BK channel activation. Based on the functional and structural information of the CTD, here we review the current molecular understanding of Ca^2+^ and Mg^2+^-dependent activation and also briefly summarize the action of other physiological ligands.

### Ca^2+^ sensors and their action

**Figure 2:**
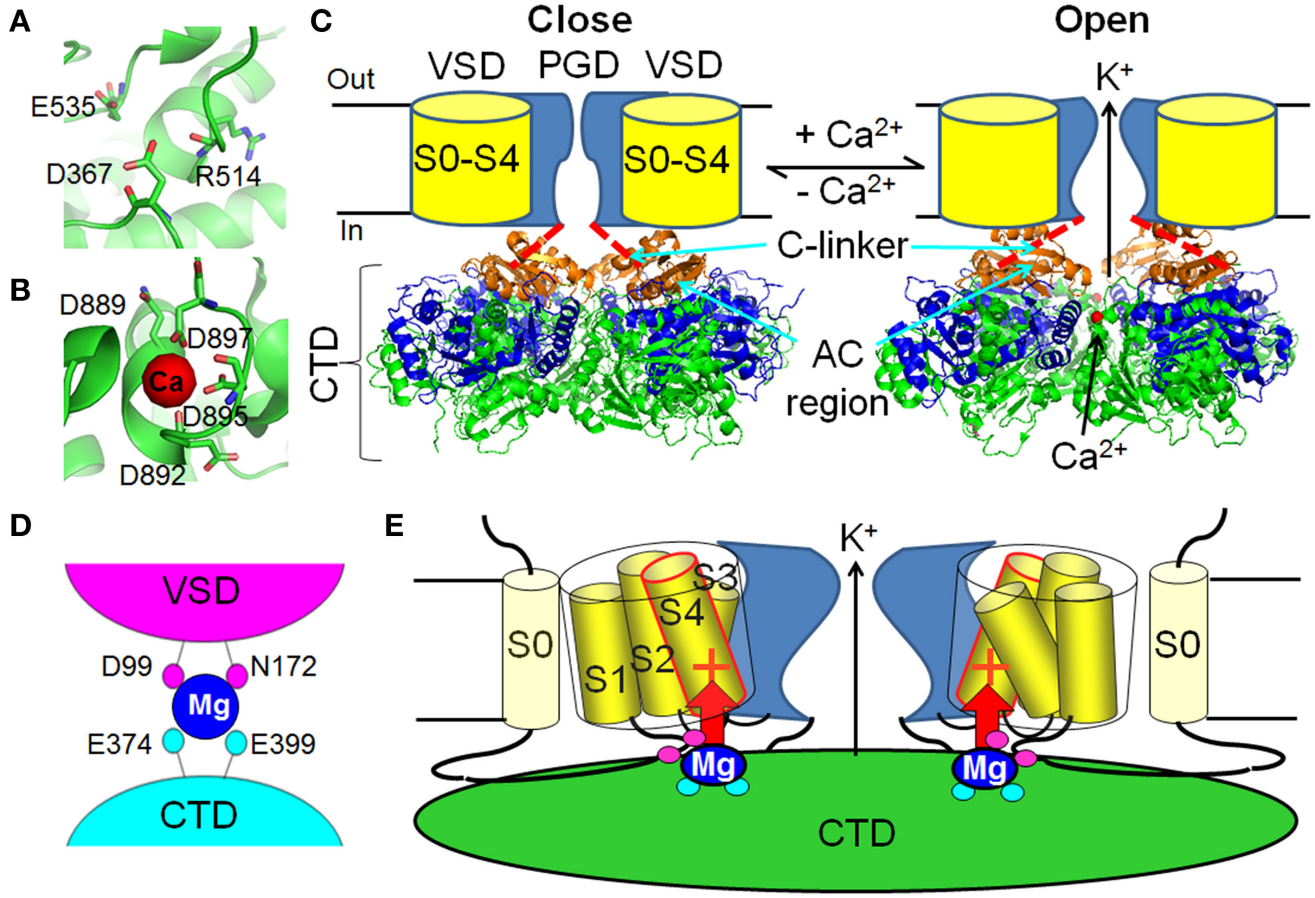
**Ca^2+^ and Mg^2+^-dependent activation of BK channels**. **(A)** The putative Ca^2+^ binding pocket in the RCK1 site (PDB ID: 3NAF). **(B)** The Ca^2+^ binding pocket in the Ca^2+^ bowl site (PDB ID: 3U6N). **(C)** Ca^2+^ binding changes the conformation of the cytosolic tail domain (CTD), which pulls the C-linker to open the activation gate of BK channels. The Ca^2+^-free (3NAF) and Ca^2+^ bound to the Ca^2+^ bowl (3U6N) CTD structures are shown in the left and right panels, respectively. One of the most dramatic Ca^2+^-induced conformational changes happens in the AC region (β A–αC, orange). The rest of the RCK1 domain is shown in blue and the RCK2 domain is shown in green. The bound Ca^2+^ in the Ca^2+^ bowl is shown as red dot in the right panel. **(D)** The low affinity Mg^2+^ binding site is composed of D99 and N172 in the voltage sensory domain (VSD) and E374 and E399 in the CTD. Magenta and cyan of these residues illustrate that D99/N172 and E374/E399 are from neighboring Slo1 subunit. **(E)** Mg^2+^ binds to the interface of the VSD and the CTD to activate BK channels through electrostatic interaction with the voltage sensor. The red + sign in S4 represents the major voltage sensing residue R213.

Intracellular Ca^2+^ binds to the CTD of BK channels to increase channel opening, typically in the range of 100 nM to 300 μM (Figures 1D,E). Electrophysiological and mutagenesis experiments have identified two Ca^2+^ high affinity binding sites for each Slo1 subunits (Figures 2A,B): one is located in the C-terminus of RCK2 domain, containing a string of Asp residues known as the “Ca^2+^ bowl” (Schreiber and Salkoff, 1997), and the other is located in RCK1 domain presumably including the side-chain carboxylates of D367 and E535, as well as the main-chain carbonyl of R514 (Shi et al., 2002; Xia et al., 2002; Zhang et al., 2010b) (Figures 1A,B).

In the recent crystal structures of the human and zebra fish BK channel CTD domains (Wu et al., 2010; Yuan et al., 2010), the Ca^2+^ bowl binding site was mapped to a short consecutive peptide containing an EF-hand-like motif at the “assembly interface” between two neighboring subunits. Within the Ca^2+^ bowl, the side chain carboxylate groups of D895 and D897 and the main chain carbonyl groups of D889 and D892 provide direct coordinates to the bound Ca^2+^ ion (Figure 2B), consistent with previous mutagenesis and biochemistry experiments (Bian et al., 2001; Braun and Sy, 2001; Bao et al., 2002, 2004). The side chain of D894 does not directly contact with Ca^2+^. Instead, it forms salt bridges with R1018 and K1030, presumably helping to stabilize the conformation of the Ca^2+^ bowl. D896, on the other hand, does not contact with other parts of the protein or Ca^2+^, and is thereby not important for Ca^2+^ binding.

Compared to the Ca^2+^ bowl site, the high affinity Ca^2+^ binding site in RCK1 domain was less well defined by the recent BK channel CTD structures. No diffraction of Ca^2+^ ion was resolved within the putative high affinity Ca^2+^ binding pocket in the RCK1 domain even when 10 or 50 mM Ca^2+^ was present during the crystallization procedures (Yuan et al., 2010). Nevertheless, the key residues (D367, E535, and R514) that have been shown to be important for Ca^2+^ sensing by functional studies (Shi et al., 2002; Xia et al., 2002; Zhang et al., 2010b) stay in close proximity in these structures, giving insights on the molecular details of this putative high affinity Ca^2+^ binding site (Figure 2A). Other residues implicated by mutational studies as being important for Ca^2+^ dependent activation, such as M513 (Bao et al., 2002) and D362 (Xia et al., 2002) do not seem to be part of the putative Ca^2+^ binding pocket as they are either chemically unfavorable (M513) or spatially far away from the binding site (D362) (Zhang et al., 2010a). It is likely that these resides indirectly contribute to Ca^2+^ binding by stabilizing the RCK1 Ca^2+^ site. Does the absence of Ca^2+^ at the RCK1 site in these structures represent artifacts during crystallization or result from the potential distortions of the Ca^2+^ site due to the absence of the entire transmembrane spanning domain that intimately interacts with the RCK1 domain (see discussion in later sections)? Further structural endeavors are thus needed to address this intriguing question.

Structural and functional studies suggest that the RCK1 Ca^2+^ site and Ca^2+^ bowl are not identical in terms of ion binding and the subsequent allosteric activation mechanism. These two sites are located, ~25Å apart, near the periphery of the gating ring (Figure 1B) with the Ca^2+^ bowl at the assembly interface between neighboring subunits and the RCK1 site in the N-lobe of the RCK1 domain (Wu et al., 2010; Yuan et al., 2010). Although both sites face toward the plasma membrane, the RCK1 site stays closer, and thus may exert more influence on the transmembrane domain including the VSD of BK channels (Figure 1B). Consistent with this scenario, voltage dependence was only observed in the binding of Ca^2+^ to the RCK1 site but not to the Ca^2+^ bowl (Sweet and Cox, 2008). The same study also discovered that the Ca^2+^ sensors exhibit different apparent Ca^2+^ binding affinity with the Ca^2+^ bowl showing higher Ca^2+^ affinity than the RCK1 site at −80 mV. Indeed, these two sites may adopt different cation coordination chemistry as evidence by their different selectivity toward various divalent cations (Zeng et al., 2005). The Ca^2+^ bowl specifically binds Ba^2+^, while the RCK1 site only senses Cd^2+^, though both sites can bind Ca^2+^ and Sr^2+^. Moreover, the two Ca^2+^ sites also exert different effects on channel kinetics (Zeng et al., 2005). The Ca^2+^ bowl mainly accelerates activation kinetics at low Ca^2+^ concentrations, while the RCK1 site influences both activation and deactivation kinetics. Considering the fact that these two Ca^2+^ sensors contribute about equally and independently to Ca^2+^ activation (Bao et al., 2002; Xia et al., 2002) with small cooperativity in activating the channel (Qian et al., 2006; Sweet and Cox, 2008), it is reasonable to assume that they operate the activation gate through different allosteric pathways. Interestingly, D369G, the human hereditary mutation associated with generalized epilepsy and paroxysmal dyskinesia (GEPD) (Du et al., 2005), enhances BK channel Ca^2+^ sensitivity specifically through the RCK1 site but not the Ca^2+^ bowl (Yang et al., 2010). The enhancing effect of the mutation was lost when the RCK1 site was destroyed, but still remained intact when the Ca^2+^ bowl site was mutated (Yang et al., 2010). Although only two amino acids away from D367 in the RCK1 site, D369G seems not directly affect Ca^2+^ binding to the RCK1 site. Instead, this mutation increases Ca^2+^ sensitivity at low Ca^2+^ concentrations by enhancing the rigidity of the N-terminal AC region (β A–αC) of the RCK1 domain (Figure 2C), a critical regulatory gating region (Krishnamoorthy et al., 2005) that couples the CTD, VSD and PGD of BK channels and exhibits most dramatic conformational changes when comparing the Ca^2+^-free and Ca^2+^-bound CTD structures (Wu et al., 2010; Yang et al., 2010; Yuan et al., 2010). With the new CTD structures, it is promising to unveil the molecular mechanism of these Ca^2+^-induced activation pathways and their potential interactions in the near future.

### Mg^2+^ sensor and its action

Under physiological conditions, millimolar intracellular Mg^2+^ can activate BK channels by shifting activation voltage to more negative ranges (Golowasch et al., 1986; Oberhauser et al., 1988). The low affinity (in millimolar range) Mg^2+^-dependent activation is independent from the high affinity (in micromolar range) Ca^2+^-dependent activation as the Mg^2+^ sensitivity remains unaltered at both zero and saturating Ca^2+^ concentrations (100 μM) (Shi and Cui, 2001). Indeed, Mg^2+^ activates the channel by binding to a low affinity divalent cation binding site distinct from the high affinity Ca^2+^ bowl and RCK1 binding sites. Electrophysiological characterization of mutations in the N-terminus of the RCK1 domain identified two acidic residues, E374 and E399 (Figure 1B), which are critical to Mg^2+^ sensing and likely to be part of the putative Mg^2+^ binding site (Shi et al., 2002; Xia et al., 2002). A comprehensive screening of all the potential oxygen-containing residues in the membrane spanning domain pinpointed D99 and N172 as the other two putative Mg^2+^ coordinates, which are located in the C-terminus of the long S0–S1 loop and the S2–S3 loop, respectively (Yang et al., 2007, 2008) (Figure 2D). Functional evidence suggests that D99 and N172 in the VSD are spatially close to E374/E399 in the RCK1 domain, thereby forming an inter-domain Mg^2+^ binding site at the interface between the VSD and the CTD (Yang et al., 2008, 2013). In the recent BK channel CTD structures (Wu et al., 2010; Yuan et al., 2010), E374 and E399 are located at the top plateau of the CTD with their carboxylate containing side chains pointing to the membrane, providing further support to the functional findings (Figure 1B).

Distinct from the Ca^2+^-dependent activation that is largely independent of the BK VSD (Horrigan and Aldrich, 2002), Mg^2+^ actually activates the channel through an electrostatic interaction with the VSD (Yang et al., 2007; Horrigan and Ma, 2008) (Figure 2E). Two lines of evidence indicate the involvement of the VSD in Mg^2+^-dependent activation. First, millimolar Mg^2+^ has no measurable effect on channel activation at negative voltages when voltage sensors are in the resting state (Yang et al., 2007; Horrigan and Ma, 2008); in contrast, 70 μM [Ca^2+^]_i_ can increase the open probability >2000-fold under similar voltages (Horrigan and Aldrich, 2002; Yang et al., 2010). This suggests that Mg^2+^ activates the channel only when the VSD stays in the activated state (Chen et al., 2011). Second, neutralization of R213, the most important voltage-sensing residue in S4, specifically eliminated Mg^2+^ sensitivity, but had no effect on Ca^2+^ sensing (Hu et al., 2003). A study further demonstrated that an electrostatic repulsion between R213 and the bound Mg^2+^ at the interface of the VSD and CTD is responsible for the activation effect of Mg^2+^ (Yang et al., 2007). This electrostatic interaction can stabilize the VSD in the activated state and alter the VSD-pore coupling (Horrigan and Ma, 2008), thereby facilitating BK channel opening.

### Other ligand sensors and their actions

**Figure 3:**
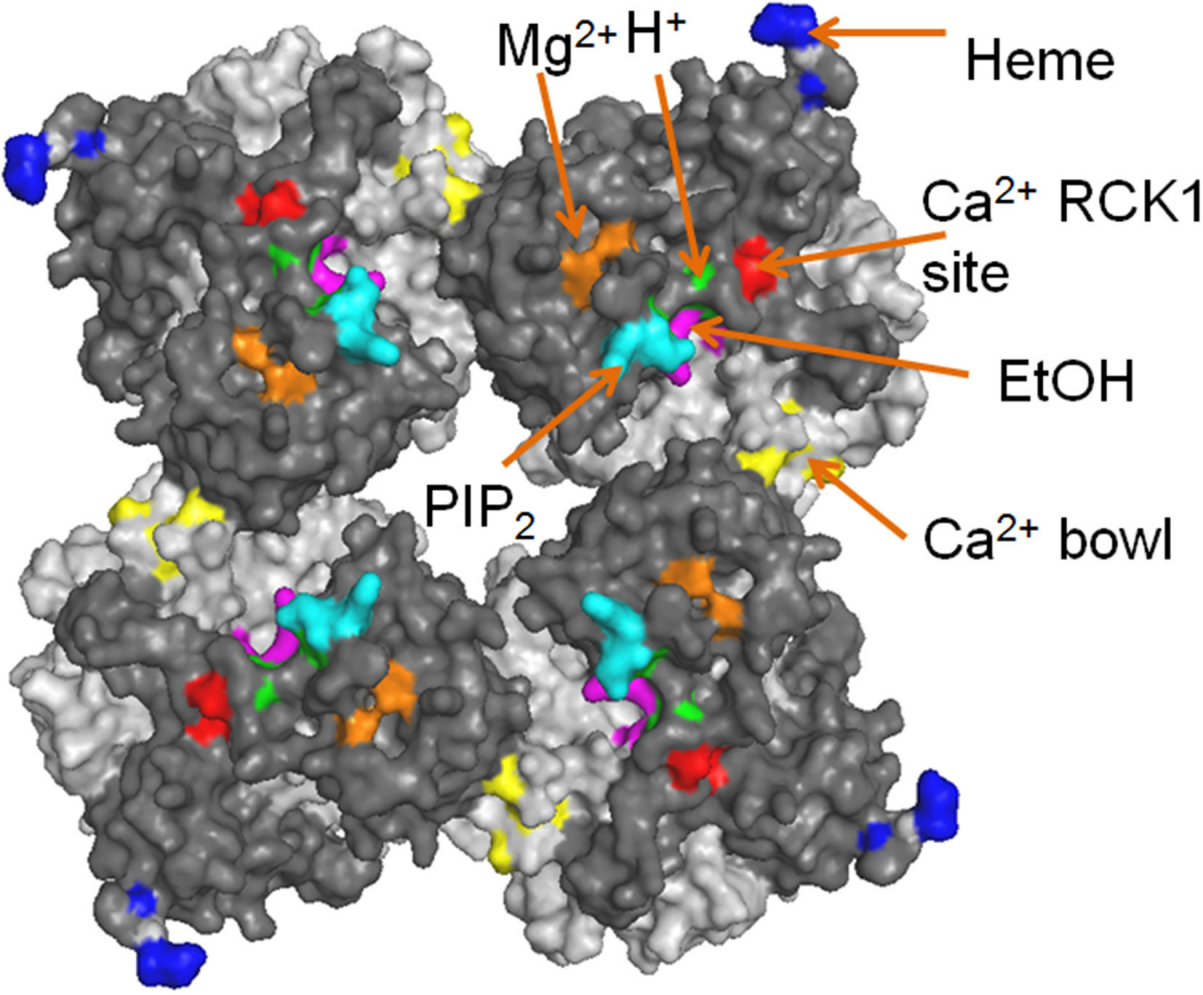
**The cytosolic tail domain (CTD) serves as chemical sensors for BK channels**. The putative binding sites for different ligands are labeled with colors. RCK1 domain and RCK2 domain are shown in dark gray and light gray, respectively. PDB ID: 3U6N.

In addition to Ca^2+^ and Mg^2+^, other intracellular ligands can also bind to the CTD domain and regulate BK channel activation (Figure 3 and Table 1). In the absence of Ca^2+^, intracellular protons have been found to be able to activate BK channels presumably by protonating the side chains of H365 and H394 (Avdonin et al., 2003; Hou et al., 2008b). As H365 is located near the RCK1 Ca^2+^ binding site, it is likely that its protonated imidazole side chains electrostatically interact with the nearby putative Ca^2+^ sensor D367 to facilitate Ca^2+^ binding. On the other hand, H394, a residue that stays further away from the RCK1 Ca^2+^ site, may indirectly affect Ca^2+^ binding and thus plays a less important role on proton sensing (Hou et al., 2008b). Interestingly, carbon monoxide (CO) also stimulates BK channels using the same sensors (Hou et al., 2008a). Mutations of H365, H394, or D367, also eliminate the CO sensitivity. These studies thus suggest that both CO and H^+^ enhance channel activation by mimicking the action of Ca^2+^ on its RCK1 sensor.

Ethanol can directly activate BK channels in isolated inside-out membrane patches (Dopico et al., 1998) in the presence of Ca^2+^ and potentiate *Caenorhabditis elegans* BK channels *in vivo* to produce alcohol intoxication by reducing excitatory neurotransmitter release (Davies et al., 2003). Further study (Liu et al., 2008c) shows that in the presence of Ca^2+^, mutations in the Ca^2+^ bowl (5D5N) or the Mg^2+^ binding site (E374A/E399A) fail to eliminate ethanol effect while mutations in the RCK1 high-affinity site (D362A/D367A) abolish ethanol inhibition of current. Based on crystallographic structure, computational modeling, mutagenesis and electrophysiology, a recent study discovered a putative ethanol recognition site in the CTD including key residues K361 and R514 (Bukiya et al., 2014). When Ca^2+^ binds to the CTD, K361 (in the middle of αA helix) likely forms hydrogen bond with the hydroxyl group of ethanol, while R514 (in the linker between αG and αH) may help to stabilize the ethanol binding pocket. It is known that ethanol cannot active BK channels in the absence of Ca^2+^. This new study gave structural explanation to this phenomenon. In the absence of Ca^2+^, the hydrogen bonding between K361 and ethanol is blocked by a nearby residue M909, and R514 swings away from the binding pocket. Both factors likely make the putative binding site inaccessible to ethanol. Consistent with this scenario, adding bulk side chains to the nearby residues E354, S357, and N358 also abolishes ethanol action, presumably by blocking ethanol accessibility. Interestingly, R514 is also involved in Ca^2+^ sensing (Zhang et al., 2010b). It is therefore plausible that Ca^2+^ binding opens up the putative ethanol binding pocket and the binding of ethanol, in turn, further facilitates Ca^2+^-dependent activation by increasing Ca^2+^ binding and/or by enhancing the allosteric coupling between Ca^2+^ binding at the RCK1 site and channel opening. By screening *C. elegans* strains with different predicted missense mutations in the *Slo1* channel from the Million Mutation Project (Thompson et al., 2013), another mutation, T352I, was found to reduce ethanol-induced activation (Davis et al., 2014). It was concluded that the T352I mutation may alter a binding site for ethanol and/or interfere with ethanol-induced conformational changes that are critical for behavioral responses to ethanol.

Lipid molecules such as PIP_2_, cholesterol and omega-3 fatty acids can also modulate BK channel activities (Braun, 2008; Vaithianathan et al., 2008; Yuan et al., 2011; Bukiya et al., 2011b; Dopico et al., 2012; Latorre and Contreras, 2013; Hoshi et al., 2013b,c,d; Tang et al., 2014). PIP_2_, a ubiquitous lipid modulator of numerous ion channels and transporters, enhances Ca^2+^-dependent gating of BK channels. Neutralizing the positively charged residue K392 and R393 in αB greatly reduced the apparent sensitivity to PIP_2_, suggesting that these two resides might be part of the putative PIP_2_ binding site (Tang et al., 2014). The localization of these residues on the top surface of RCK1 domain is consistent with this hypothesis, which potentially allows electrostatic interactions between the positively charged residues and the negatively charged PIP_2_ head group in the inner leaflet of the plasma membrane (Figure 3). Interestingly, the PIP_2_ effect was only apparent when potent PIP_2_ depleting reagents were applied, suggesting that PIP_2_ might tightly bind to BK channels and/or the intimate interactions between the CTD and the membrane spanning domain of BK channels create a physical barrier to limit the free diffusion of this highly charged lipid species. Different from PIP_2_, Omega-3 fatty acids were recently discovered to act on BK channels through Y318 at the C-terminus of S6 segment (Hoshi et al., 2013d). These lipids potentiate BK current in the presence of auxiliary β 1 subunit and lower blood pressure in mice (Hoshi et al., 2013c), thereby providing a molecular mechanism to explain potential health benefits of omega-3 fatty acids on regulating blood pressure. In contrast, cholesterol inhibits BK channel activity (Bolotina et al., 1989; Bregestovski and Bolotina, 1989; Dopico et al., 2012). Cholesterol either works directly on BK channel complexes and/or alters BK channel activity indirectly by modulating membrane lipids or lipid-channel interfaces (Bukiya et al., 2011a,b; Dopico et al., 2012; Singh et al., 2012a).

Heme inhibits BK channel activity by binding to the CTD with high affinity (IC_50_ = ~70 nM) (Tang et al., 2003; Horrigan et al., 2005). Bioinformatics predication and the subsequent functional characterization identified the sequence “CKACH” in the N-terminus of the RCK1-RCK2 linker to be responsible for heme binding (Tang et al., 2003). A comprehensive analysis of heme effects using the HA allosteric model (Horrigan and Aldrich, 2002) suggested that heme exerts its apparent inhibitory effect by increasing open probability (Po) at negative voltages and reducing Po at more positive voltages (Horrigan et al., 2005). The binding of heme to the RCK1-RCK2 linker segment that is located at the periphery of the CTD domain (Figure 3) may impede the gating ring conformational changes and the CTD-VSD interaction that normally accompanies the activation of BK channels.

It is worth noting that, except for the Ca^2+^ bowl in the RCK2 domain, all the known chemical sensors are located on or close to the top surface of the RCK1 domain that faces the membrane or the membrane-spanning domain of the channel (Figure 3). Since these sites are sensors of cytoplasmic ligands, they are exposed to aqueous solution. It is interesting that all the “activating” sensors in the RCK1 domain are clustered at the center of the gating ring, while the “inhibiting” heme sensor is located at the periphery. Does this design reflect a coincidence or an evolutionary advantage in regulating BK channel activation? How do these sensors interact with each other? Do they have any cooperativity? Answers to these questions will further our understanding of the mechanisms of BK channel activation.

## The pore-gate domain controls K^+^ permeation of BK channels

Free energies provided by membrane voltage and intracellular ligand binding ultimately alters the PGD to open to K^+^ flux across the membrane. The PGD domain, comprising S5–S6 segments, forms the center of a BK channel, where the ion selection and permeation occur. Like most of other K^+^ channels, a short peptide including the signature “GYG” sequence from four Slo1 subunits form the ion selectivity filter of a BK channel, which separates the external and internal aqueous solution and selectively permits K^+^ ions to go through (Doyle et al., 1998). Four S6 helices (equivalent to the inner helix in 2-TM K^+^ channels) from each of Slo1 subunit form the central ion pathway. Despite these similarities, BK channels exhibit a number of functional and structural features that distinguish them from other Kv channels.

First, BK channels have the largest unitary conductance of all K^+^ channels. Its large conductance is partly derived from two clusters of acidic residues that are located at the intracellular and extracellular entrances of the K^+^ permeation pathway. D261 in the extracellular entrance contribute to ~18% of BK channel unitary conductance for the inward K^+^ current (Carvacho et al., 2008), while E321 and E324, which are located in the cytosolic end of the S6 segment, form a ring of 8 negative charges and contribute up to 50% of BK unitary conductance for the outward K^+^ current (Brelidze et al., 2003; Nimigean et al., 2003). These clusters of negative charges thus serve as electrostatic traps to attract and concentrate local K^+^ concentration to enhance BK unitary conductance (Brelidze et al., 2003; Nimigean et al., 2003; Carvacho et al., 2008). Nevertheless, these negative charges at the extracellular and intracellular entrances of K^+^ permeation pathway only account for part of the large single channel conductance of BK channels. Other structural features specific to BK channels, including the larger negative electrostatic potential inside the pore and the wider entrance to the inner vestibule, may also contribute to BK channels' large conductance (discussed below) (Nimigean et al., 2003; Li and Aldrich, 2004; Brelidze and Magleby, 2005; Carvacho et al., 2008; Geng et al., 2011).

Second, BK channels have a much larger inner vestibule with a wide cytosolic entrance compared to most of K^+^ channels. Chemicals with various sizes and properties were used to probe the size of the central cavity and its cytosolic entrance (Li and Aldrich, 2004; Brelidze and Magleby, 2005; Wilkens and Aldrich, 2006). Smaller size quaternary ammoniums (QA) such as tetrabutylammonium (TBA) can have relatively free access to the inner vestibule independent of the states the activation gate. These QAs show much faster blocking and unblocking kinetics in BK channels than in other Kv channels, indicating BK channels have an enlarged inner vestibule and broader cytosolic entrance (Li and Aldrich, 2004; Wilkens and Aldrich, 2006). Based on the changes of the K^+^ diffusion rate from bulk intracellular solution to the central cavity due to interference by sucrose, the cytosolic mouth of BK channel pore when open was estimated to be twice (~16–20Å) as large as that of the Shaker K^+^ channel (Brelidze and Magleby, 2005). Consistent with this estimation, recent cysteine substitution and modification studies of S6 with different MTS reagents showed that modification can occur even when the channel is in closed states (Geng et al., 2011; Zhou et al., 2011). The cytosolic opening of the central cavity at the level of the C-terminus of S6 (around E321 and E324) is at least 13–18 Å in diameter, which allows MTS reagents to go through and modify the cysteine residues inside the central cavity (Zhou et al., 2011) or the cysteine residues at the cytosolic entrance to alter outward single channel conductance (Geng et al., 2011). All these results suggest that BK channel S6 lacks the cytosolic activation gate around the “bundle crossing” in canonical K^+^ channels, where the hydrophobic residues at the C-terminus of the four inner helices form a tight seal to restrict K^+^ ion flux when these channels are closed (Hille et al., 1999). Instead, the activation gate of BK channels is likely near or within the selectivity filter, a design that is also observed in other ligand-gated ion channels such as CNG (cyclic nucleotide-gated) channels (Flynn and Zagotta, 2001) and SK (small conductance, Ca^2+^-activated K^+^) channels (Bruening-Wright et al., 2007). The interaction between permeating thallium ion (Tl^+^) and the selectivity filter altering BK channel activation further supports this scenario (Piskorowski and Aldrich, 2006).

**Figure 4:**
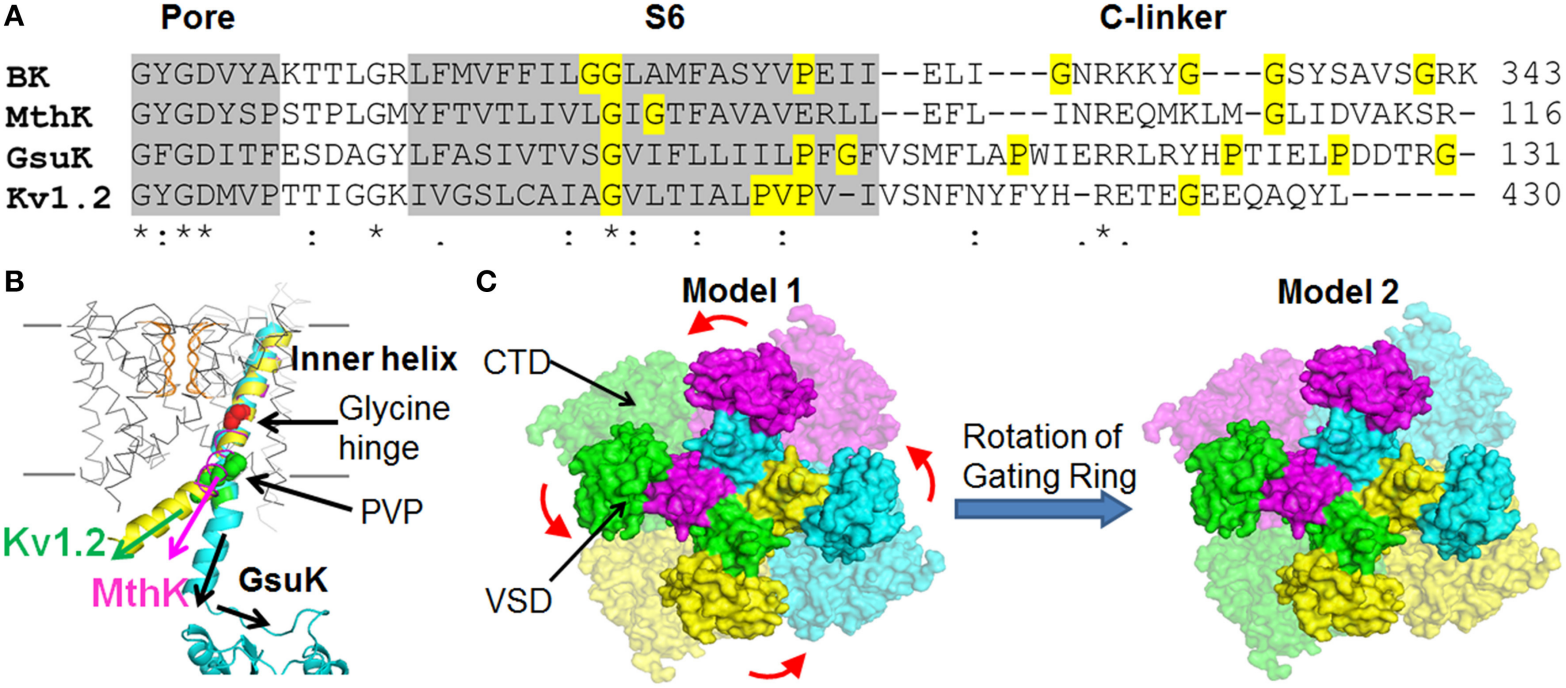
**Assembly of the BK channel structural domains**. **(A)** Sequence alignment of the inner helices and the C-linkers of different K^+^ channels. Residues that can increase peptide flexibility and alter α helix orientation (i.e., Glycine and Proline) are highlighted in yellow. **(B)** The C-termini of inner helix and the C-linkers of different K^+^ channels may point to different directions. The PGDs of MthK (PDB ID: 1LNQ), Kv1.2 (PDB ID: 2R9R) and GsuK (PDB ID: 4GX5) channels are superimposed at the selectivity filter by using UCSF Chimera. **(C)** The relative assembly of the VSD and the CTD in BK channels might be different from the homology model shown in Figure 1B with a relative ~90° angular rotation about the central axis between the PGD and CTD.

Third, the orientation of the pore-lining residues in BK channels is different from those of Kv channels. Cysteine substitution and modification studies of the BK S6 demonstrated that A313, A316, and S317 are facing to the inner pore, while the corresponding residues in Shaker K^+^ channels tend to face away from the aqueous environment (Zhou et al., 2011). Therefore, a relative rotation of the S6 has to occur to account for this experimental observation. One possible cause of this rotation may derive from the two consecutive glycine residues (G310 and G311) in BK channel S6 (Figure 4A). An additional glycine residue (G310) may make S6 more flexible around the highly conserved “Glycine hinge” region in Kv channels, thereby rearranging the orientations of the residues downstream of this di-glycine hinge.

The forth unique feature of the BK PGD is that the movement of the pore-lining S6 helix of BK channels is different from that of canonical K^+^ channels. Although BK channel pore has an enlarged intracellular entrance, state-dependent blockade by a synthetic Shaker ball peptide (ShBP) suggests that the S6 segment corresponding to this entrance indeed moves during gating to restrict the entry of bulky ShBP but not smaller K^+^ and QA ions (Li and Aldrich, 2006). Structural and functional studies of K^+^ channels suggest that the highly conserved glycine hinge in the middle (Jiang et al., 2002b) and/or the Pro-Val-Pro (PVP motif) at the C-terminus of the pore-lining S6 helix (Webster et al., 2004) are two critical pivot points for the movement of the activation gate. Ala mutations of the double glycine resides (Magidovich and Yifrach, 2004) significantly hinders channel activation, suggesting that the flexibility around the di-Gly hinge is critical for BK channel gating. Indeed, the recent mutagenesis, cysteine modification, histidine protonation and pharmacological studies of BK channels show that multiple pore residues (L312, A313, M314, and A316) downstream of the di-glycine hinge reorient their side chains during channel gating (Chen et al., 2014). Remarkably, charged or polar side-chain substitutions at each of the sites resulted in constitutively opened mutant channels that largely or completely loss voltage and Ca^2+^ dependence, presumably by exposing the hydrophilic side chains to the aqueous environment of the pore to reduce their side-chain solvation energy. Based on the fact that multiple pore residues in BK displayed side-chain hydrophilicity-dependent constitutive openness, it is proposed that BK channel opening involves structural rearrangement of the deep-pore region.

## Assembly of the BK channel modular domains

Increasing structural and functional information is available on the individual PGD, VSD, and CTD and their intimate interactions. However, how these domains spatially assemble to form a quaternary structure of a functional tetrameric BK channel is still unclear largely owing to the lack of a full-length atomic structure. Thus, the most widely used method to address this problem is to construct a BK channel structure through homology modeling (Figure 1B). With the crystal structures of Kv1.2–Kv2.1 chimera channel (Long et al., 2007) and the BK CTD (Wu et al., 2010; Yuan et al., 2010), a homology model of BK channels can be built using the MthK structure, which contains a homologous PGD and CTD, as a template (Jiang et al., 2002a,b). A VSD structure was included in the BK channel model by superimposing the PGDs of the Kv1.2–Kv2.1 chimera channel and the MthK channel; while the gating ring of MthK was replaced by the BK channel CTD structure. Despite the fact that the VSD lacks S0 and the long S0–S1 linker, the overall structure of this homology model is consistent with most of functional findings in BK channels. According to this model, the membrane spanning domain and the CTD gating ring are closely packed to each other with the VSD residing on the top surface of RCK1 domain along the four-fold (central) axis. The close proximity of the CTD to the membrane spanning domain is consistent with the following evidence. First, the CTD and the membrane spanning domain were tightly packed to each other in a cryo-electron microscopy structure of the full-length BK channel (Wang and Sigworth, 2009). Second, the top plateau of the RCK1 domain, especially αB helix might move toward the membrane-spanning domain along the central axis when compared the Ca^2+^-bound CTD structure (3U6N) with the Ca^2+^-free CTD structure (3NAF) (Wu et al., 2010; Yuan et al., 2012) (Figure 2C). Third, four residues from the VSD and the CTD are in close proximity and form an inter-domain Mg^2+^ binding site (Yang et al., 2008) (Figure 2D). Fourth, Mg^2+^ or the charges around the Mg^2+^ binding site in the CTD can electrostatically interact with R213 in S4 of VSD (Hu et al., 2003) (Figure 2E). Fifth, introducing charges to N172 in the VSD and E399 in the CTD creates electrostatic interactions that affect the voltage- and Ca^2+^-dependent activation of BK channels (Yang et al., 2013). Last but not least, K392 and R393 on the top surface of the CTD can electrostatically interact with negatively charged PIP_2_ molecules on the inner leaflet of the plasma membrane (Tang et al., 2014) (Figure 3).

While the relative longitudinal packing of the VSD and the CTD along the four-fold central axis in the homology model is consistent with experimental results, the relative angular positions about the central axis between the PGD and CTD of the same subunit are not defined in the BK channel models (Figure 4C). First of all, the 17-amino acid peptide C-linker between the PGD and CTD was not resolved in the template MthK structure (Jiang et al., 2002a,b), leaving some uncertainties in the assignment of the PGD and the CTD to the same subunit. Secondly, although both MthK and BK channels contain two glycine residues at the “Glycine hinge” in S6 of the PGD the localization of the Glycine residues differs between the two channels (Figure 4A). The C-terminus of S6 in the MthK channel also lacks a Proline, the helix-breaking residue, compared to BK channels. In addition, the C-linker between S6 and the CTD of the BK channel differs from that of the MthK channel in both length and amino acid sequences. This series of differences suggest that the CTD angular position relative to the PGD can differ between MthK and BK channels. Interestingly, recently published crystal structures of a full-length GsuK channel, a RCK-containing, multi-ligand gated K^+^ channel from bacteria *Geobacter sulfurreducens*, show a ~50 degree of counterclockwise rotation of the CTD relative to the PGD as compared to the MthK structure (Kong et al., 2012). The “glycine hinge” residues and the C-terminal Proline residue in S6 are not conserved between the GsuK and MthK channels (Figure 4A). In addition, the C-linker of the GsuK channel is six amino acids longer than that of the MthK channel. Containing three proline residues and one glycine residue, the GsuK C-linker is also flexible as evident in the crystal structure (Figure 4B). These properties might contribute to this rotation.

Based on the study of the Mg^2+^ binding site (Yang et al., 2008), which is composed of D99/N172 from the VSD and E374/E399 from the CTD (Figure 2D), it was proposed that the VSD of each subunit is aligned with the CTD from a neighboring subunit. This alignment requires relative angular positions between the CTD and PGD differing from the homology model based on MthK structure (Figure 4C). While individual mutations D99R, N172R, E374R, and E399C abolished Mg^2+^ sensitivity of the homotetrameric BK channels, the heterotetrameric channels resulting from the co-expression of the BK channel subunits containing D99R in the VSD and E374R in the CTD, respectively, still retained partial Mg^2+^ sensitivity. The retention of Mg^2+^ sensitivity can only be explained by the BK homology model 2 (Figure 4C), in which D99/N172 in the VSD of one subunit and E374/E399 in the CTD from the neighboring subunit form an intersubunit Mg^2+^ binding site. In this way, one or two out of four Mg^2+^ binding sites could remain intact in some of the heterotetrameric channels. The experimental results could be nicely fitted with a binomial distribution of the mixed mutant channels. On the other hand, the BK homology model based on the MthK structure predicts that all four Mg^2+^ binding residues are from the same Slo1 subunit to form an intrasubunit Mg^2+^ site. Since one single mutation is sufficient to eliminate Mg^2+^ binding, none of the heterotetrameric channels could retain intact Mg^2+^ binding site based on this model. Consistent with this alignment, it is recently reported (Zhang et al., 2014) that mutation E219R in S4 and E321/E324 in the cytosolic side of S6 of a neighboring subunit engage in electrostatic interactions to alter voltage and Ca^2+^ dependent activation. These results suggest that the BK channel S6 may bend at the inner vestibule as compared to the structure of Kv1.2/Kv2.1 (Figure 4B), directing the downstream CTD to an angular position shown in the homology model 2 (Figure 4C). This arrangement may also explain why BK channels have large openings at the inner vestibule even when the channel is closed.

## Allosteric coupling between the sensors and the pore-gate

The VSD and CTD in BK channels sense voltage and intracellular signaling molecules and open the channel gate in the PGD by electromechanical and chemomechanical couplings between the PGD and the VSD and CTD, respectively (Figure 1C). Voltage and Ca^2+^ activate BK channels mainly by destabilizing the closed state with small effects on stabilizing the activated state of the PGD (Geng and Magleby, 2014). Various allosteric models have been developed to describe Ca^2+^- and voltage-dependent BK channel gating based on the analysis of single channel kinetics and macroscopic current (McManus and Magleby, 1991; Cox et al., 1997; Horrigan et al., 1999; Rothberg and Magleby, 1999, 2000; Cui and Aldrich, 2000; Horrigan and Aldrich, 2002; Shelley et al., 2010). In a model that integrates both Ca^2+^ and voltage dependent activation (Figure 1C) (Horrigan and Aldrich, 2002), three structural domains, the PGD, VSD and CTD, undergo separate conformational changes but also allosterically coupled to each other, reflecting the modular design of BK channels. The BK channel activation gate can open in the absence of voltage sensor activation and Ca^2+^ binding with an intrinsic open probability of ~10^−7^ (Horrigan et al., 1999; Cui and Aldrich, 2000). On the other hand, voltage sensor activation and Ca^2+^ binding can enhance channel opening in a relatively independent fashion. In the absence of Ca^2+^ binding, extreme depolarization (> +110 mV) enhances channel open probability when the voltage sensor moves from resting state to activated state (Figures 1D–G) (Cui et al., 1997). Similarly, saturating Ca^2+^ increases BK channel open probability by four orders of magnitude from ~10^−7^–10^−3^ when the voltage sensors are at the resting state (Horrigan and Aldrich, 2002; Yang et al., 2010), indicating a strong interaction between Ca^2+^ binding and channel opening. A weak interaction between voltage sensor activation and Ca^2+^ binding also exists (Horrigan and Aldrich, 2002; Sweet and Cox, 2008), though the mechanism of this interaction is less clear.

The allosteric coupling between the CTD and the PGD of BK channels is mainly mediated by the C-linker that covalently connects these two domains (Figure 2C). A comparison between the CTD crystal structures with and without Ca^2+^-bound to the Ca^2+^ bowl suggests that the N-terminal lobe of RCK1 domain undergoes most dramatic conformational changes upon Ca^2+^ binding to the Ca^2+^ Bowl compared to other regions in the CTD (Wu et al., 2010; Yuan et al., 2010). The conformational changes are distinct from the conformation changes of the MthK channel gating ring, which reduces its height and expands its diameter upon Ca^2+^ binding (Jiang et al., 2002a,b). In BK channels, the N-terminal lobe of RCK1 resides on the top layer of the gating ring directly facing the membrane and covalently connecting to the PGD through the C-linker. Upon Ca^2+^ binding, this lobe rotates relative to RCK2 domains as a rigid body, resulting in an expansion of the top layer of the gating ring (from a diameter of 81–93 Å measured at Cα atoms of the N-terminal residues of RCK1, K343). Opening like the petals of a flower, this mechanical force will directly pull the C-linker to open the PGD. This mechanical model is consistent with an early functional study by the Magleby group, who discovered that the length of the C-linker is critical to channel activation. Shortening the C-linker enhances channel activity and lengthening the linkers decreases channel activity, both in the presence and absence of intracellular Ca^2+^ (Niu et al., 2004). Therefore, the C-linker might serve as a passive spring to control BK channel gating. Interestingly, a recent functional study demonstrated that BK channel openers, such as Cym04 and NS1619, activate BK channels by functionally interacting with the C-linker, thereby mimicking site-specific shortening of the C-linker (Gessner et al., 2012).

As discussed above, the free energy of Ca^2+^ binding to the Ca^2+^ bowl and the RCK1 site may propagate via different pathways to open the activation gate. As the C-linker provides the only covalent linkage between the CTD and the PGD, it is conceivable that these two separate Ca^2+^-activation pathways may converge at the C-linker to operate the gate. Nevertheless, the non-covalent domain-domain interactions among the CTD, VSD and the PGD may provide additional pathways to differentially mediate Ca^2+^ dependent activation originated from the Ca^2+^ bowl and the RCK1 site. It is unclear whether the possible modes of the quaternary assembly of BK channels (Figure 4C) have any impact on the coupling of the Ca^2+^ sensors to the activation gate. Further experiments are needed to address this question.

The molecular mechanism of electromechanical coupling between the VSD and the PGD in BK channels is less well understood compared to other Kv channels. In Kv channels, the S4–S5 linker directly contact with the C-terminus of S6 to transduce the energy of the VSD movement to gate opening (Lu et al., 2001). In a recent study, the Arginine mutation of E219 in the lower S4 segment was shown to have an electrostatic interaction with E321 and E324 at the C-terminus of S6 (Zhang et al., 2014), suggesting that BK channels may use the similar mechanism to couple the VSD to its PGD as Kv channels. Nevertheless, this electrostatic interaction is rather long range compared to the short-range hydrophobic interactions observed in Kv channels. Given that the conformation of BK S6 might be different from that of Kv channels, it is therefore very likely other coupling sites and mechanism also exist to couple the VSD to the PGD.

Although the coupling between the VSD and CTD is relatively weak (Horrigan and Aldrich, 2002), the interaction between these two domains does exist and is important in controlling BK channel activation. A well-understood example is the interactions among the residues around the Mg^2+^ binding resides in both the CTD and VSD that can affect VSD activation and the intrinsic open probability of the activation gate (Yang et al., 2006, 2007, 2013). Considering the clusters of the ligand binding sites that are located at the interface between the CTD and VSD (Figure 3), it is conceivable that more direct interactions between these two sensory modules may exist and mediate their synergy in activating the PGD.

## Author contributions

All authors contributed to the writing, revising, and approval of the manuscript.

### Conflict of interest statement

The authors declare that the research was conducted in the absence of any commercial or financial relationships that could be construed as a potential conflict of interest.
